# Vertebral compression fracture with diffuse idiopathic skeletal hyperostosis

**DOI:** 10.1002/jgf2.495

**Published:** 2021-09-02

**Authors:** Mayu Uemoto, Junki Mizumoto

**Affiliations:** ^1^ Department of Internal and Family Medicine Takamatsu Heiwa Hospital Kagawa Japan; ^2^ Department of Medical Education Studies International Research Center for Medical Education Graduate School of Medicine The University of Tokyo Tokyo Japan

**Keywords:** emergency medicine, family medicine

## Abstract

A 90‐year‐old Japanese man presented with back pain after falling. Imaging tests revealed compression fracture of the lumber vertebrae with diffuse idiopathic skeletal hyperostosis (DISH), and surgical intervention was performed. Back pain is common in primary care setting, and primary care physicians should recognize this condition well.

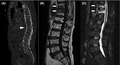

A 90‐year‐old Japanese man presented to the emergency department complaining of backache and difficulty in walking after falling on his buttocks. On physical examination, severe tenderness over the lumbar vertebrae was found. There were no neurological deficits or pain in the pelvis or femurs. Compression fracture of the lumbar vertebrae was suspected. A computed tomography (CT) scan was performed to evaluate structural abnormalities, and injury of ligament with ossification with diffuse idiopathic skeletal hyperostosis (DISH) was detected (Figure 1A). Magnetic resonance imaging (MRI) revealed a low‐intensity area on T1‐weighted images and a high‐intensity area on T2‐weighted images of the 11th and 12th thoracic vertebrae (Figure 1B,C). The diagnosis of vertebral compression fracture with anterior tension band injury was made. Thoracolumbar AOSpine Injury Score was 7 (type B3, N0, M0), and surgical intervention was strongly recommended. The patient was admitted to the hospital, and surgical posterior fusion of the spine was performed in accordance with the classification and previous research.^1^ He was transferred to a rehabilitation hospital on postoperative day 30 with no neurological problems.

**FIGURE 1:**
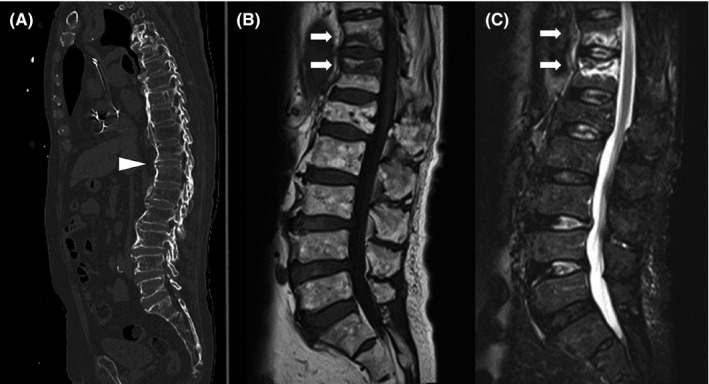
A, Fracture of the bone growth plate between the 11th and 12th thoracic vertebrae in a patient with DISH on a CT scan (arrowhead). B, Low‐intensity area in the 11th and 12th thoracic vertebrae on T1‐weighted images (arrow). C, High‐intensity area on the 11th and 12th thoracic vertebrae on T2‐weighted images (arrow)

DISH is a noninflammatory disease where spinal ligaments and entheses become calcified.^2^ Vertebral compression fracture is usually treated conservatively, except for rupture fractures and fractures causing neurological deficits. Compression fractures in DISH are another exception and require surgical treatment to prevent the delayed development of neurological disorders.^3^ Thoracolumbar AOSpine Injury Score,^4^ according to which fracture cases with 6 points or more were recommended for surgical operation, rates anterior tension band injury 7 points. This means that compression fracture in DISH should be treated surgically, regardless of the presence or absence of neurological deficits immediately at the time of initial examination.

Back pain is one of the most common symptoms in a primary care setting.^5^ Vertebral compression fracture is particularly common in older patients, and minimal trauma may lead to this fracture. Although it is often diagnosed using x‐ray,^6^ the detailed structural deformation may not be revealed until a CT or MRI scan is performed. When patients with previously known DISH or with risk factors of developing DISH (eg, older age, obesity, gout, or type 2 diabetes mellitus)^7^ present with back pain or have an episode of falling, primary care physicians should refer the patient to specialists and ask them to perform a CT or MRI scan to evaluate whether surgical intervention is needed.

In summary, vertebral compression fracture in patients with DISH should be treated surgically because of the potential for neurological complications. Primary care physicians should remember which types of fracture may be candidates for surgery and refer patients with suspected vertebral compression fractures with DISH to specialists for further evaluations.

## CONFLICT OF INTEREST

The authors have stated explicitly that there are no conflicts of interest in connection with this article.

## INFORMED CONSENT

Informed written consent was obtained from the patient for publication of this report and any accompanying images.
